# Does Glyphosate Affect the Human Microbiota?

**DOI:** 10.3390/life12050707

**Published:** 2022-05-09

**Authors:** Pere Puigbò, Lyydia I. Leino, Miia J. Rainio, Kari Saikkonen, Irma Saloniemi, Marjo Helander

**Affiliations:** 1 Department of Biology, University of Turku, 20500 Turku, Finland; lyirle@utu.fi (L.I.L.); miikoi@utu.fi (M.J.R.); irma.saloniemi@utu.fi (I.S.); helander@utu.fi (M.H.); 2 Nutrition and Health Unit, Eurecat Technology Centre of Catalonia, 43204 Reus, Catalonia, Spain; 3 Department of Biochemistry and Biotechnology, Rovira i Virgili University, 43007 Tarragona, Catalonia, Spain; 4 Biodiversity Unit, University of Turku, 20500 Turku, Finland; karisaik@utu.fi

**Keywords:** glyphosate, herbicide, sensitivity, human microbiota, multi-antibiotic resistance

## Abstract

Glyphosate is the world’s most widely used agrochemical. Its use in agriculture and gardening has been proclaimed safe because humans and other animals do not have the target enzyme 5-enolpyruvylshikimate-3-phosphate synthase (EPSPS). However, increasing numbers of studies have demonstrated risks to humans and animals because the shikimate metabolic pathway is present in many microbes. Here, we assess the potential effect of glyphosate on healthy human microbiota. Our results demonstrate that more than one-half of human microbiome are intrinsically sensitive to glyphosate. However, further empirical studies are needed to determine the effect of glyphosate on healthy human microbiota.

## 1. Introduction

**Figure 1:**
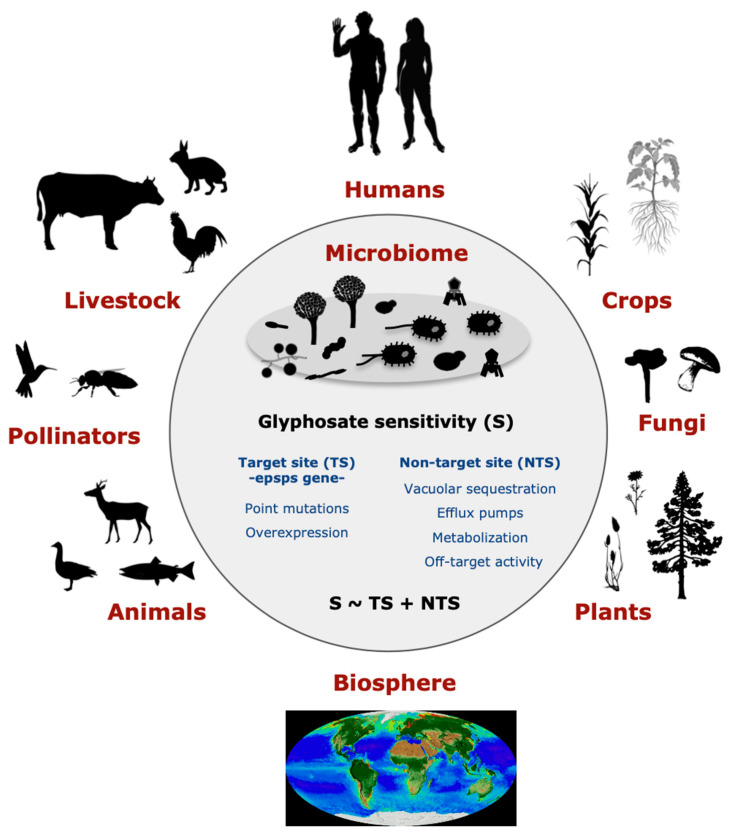
Potential effects of glyphosate on the microbiome may have an impact on environmental health, human health, and sustainability. Glyphosate may influence healthy microbiota due to its action on the EPSPS enzyme, glyphosate target site (TS), and other non-target site (NTS) mechanisms. A healthy microbiota presents diverse species that are either sensitive or resistant to the herbicide. Thus, the heavy use of glyphosate-based products may lead to microbial dysbiosis by enhancing the spread of resistant and fast-evolving bacteria and selecting against sensitive ones. The consequences of this imbalance in the microbiota may have a wide-ranging ecological impact.

Glyphosate (N-(phosphonomethyl)glycine) is globally the most commonly used herbicide in agriculture, horticulture, silviculture, recreational areas, and home gardens [1,2]. The popularization of glyphosate-based herbicides (GBHs) has been associated with an increased detection of glyphosate and its by-product aminomethylphosphonic acid (AMPA) in soil and water [3,4,5]. Here, we perceive the risk that glyphosate may modulate microbes that are essential to human well-being because the targeted shikimate pathway is present in many microbes [6,7,8,9,10,11,12]. The herbicide inactivates the central enzyme 5-enolpyruvylshikimate-3-phosphate synthase (EPSPS), an almos-universal enzyme in plants, fungi, and prokaryotes, for the synthesis of three aromatic amino acids [13,14]. Some species have evolved a variety of resistance mechanisms to glyphosate (Figure 1), including target-site resistance (TSR), i.e., the direct effect of glyphosate on the EPSPS enzyme [6], and non-target-site resistance (NTSR) [15]. TSR adaptations can be determined based on amino acid biomarkers in the EPSPS active site that classify the enzyme as potentially sensitive or resistant to glyphosate [6]. Currently, four classes of EPSPS enzymes have been recognized as potentially sensitive (class I) or resistant (class II–IV) and can be determined based on bioinformatic methods. NTSR mechanisms may reduce the sensitivity of organisms to glyphosate by efflux pumps and the overexpression of the *epsps* gene [16]. Alternatively, they may increase sensitivity via the mitochondrial transport chain [17].

## 2. Materials and Methods

### 2.1. Dataset of EPSPS Proteins

A dataset of the 732 bacterial genomes was obtained from the Human Microbiome Project (HMP) [18]. Genomes were mapped through BLAST searches onto the COG0128 from the database of Cluster of Orthologous Groups (COG) [19] to identify EPSPS proteins. The dataset is available in Supplementary Table S1. 

### 2.2. Potential Sensitivity and Resistance to Glyphosate

Glyphosate targets the EPSPS enzyme by competing for the binding site with phosphoenol pyruvate (PEP) [20]. The enzyme has been categorized into four classes based on its potential sensitivity to glyphosate [6,21]. Classes I, II, and IV were determined based on the presence and absence of amino acid markers in active sites, whereas Class III was categorized based on a series of motifs. Biomarkers were identified based on amino acid residues in the EPSPS of *Vibrio cholerae* (Class I), *Coxiella burnetii* (Class II), *Brevundimonas vesicularis* (Class III), and *Streptomyces davawensis* (Class IV). These reference sequences are used on the web server http://ppuigbo.me/programs/EPSPSClass/ (accessed on 1 December 2021) to determine the intrinsic sensitivity of EPSPS enzymes.

## 3. Results and Discussion

Recently, we combined closely related bacterial species and different strains within species to identify changes in their sensitivity to glyphosate [7] under the microevolutionary perspective of Alignable Tight Genomic Clusters (ATGC) [22]. The study of the EPSPS enzyme showed that phylogenetic groups and bacterial lifestyle are key factors determining the intrinsic sensitivity to glyphosate, possibly resulting from thicker cell walls in addition to differences in EPSPS type [7,8]. Specifically, Firmicutes were significantly more resistant to glyphosate than the most sensitive Proteobacteria and Actinobacteria groups. Moreover, a bacterial lifestyle was strongly associated with sensitivity, because facultative host-associated and parasitic bacteria are more sensitive to the herbicide than free-living bacteria. However, Van Bruggen et al. showed that pathogens are generally less sensitive to glyphosate than host-associated and free-living bacteria, by analyzing literature data on minimal inhibitory concentrations for a large number of bacteria [8]. The microevolutionary analysis further revealed that bacteria may easily become resistant to glyphosate through small changes in the EPSPS active site in the short evolutionary time of ATGC with non-synonymous mutations and horizontal gene transfers [7]. Thus, the heavy use of glyphosate may have a strong impact on the species diversity and composition of microbial communities via (1) the purifying selection against sensitive bacteria, (2) the rapid adaptation of some bacterial groups to become resistant to glyphosate, and (3) the potential glyphosate-related multidrug resistance in bacteria [7,12,23].

Humans may be exposed to glyphosate directly when applying glyphosate-based herbicides or indirectly via drinking water and foodstuffs containing glyphosate residues [24,25]. In traditional agricultural practices, glyphosate-based herbicides are applied before planting and after harvest, but in genetically modified glyphosate-resistant crops, they can be used during the growing season. In addition, glyphosate-based herbicides are commonly used to desiccate traditional grain and seed crops before harvest. These practices include the risks of inhalation and skin exposure to the applicator. Residues in ingested products may lead to the exposure of human gastrointestinal and urogenital systems’ microbiota to glyphosate and its metabolites. Human cells are presumably not directly affected by glyphosate due to the lack of the EPSPS enzyme. However, the effect of glyphosate on the host-associated microbiota has been suggested in several studies of insects, plants, and mammals [7,9,10,26,27,28]. In our previous study [6], we performed a survey of 890 EPSPS sequences to evaluate the potential sensitivity to glyphosate in 101 common human gut bacterial species [29]. We found that 54% of most common gut bacterial species are intrinsically sensitive to glyphosate, i.e., these species present amino acid biomarkers that determine the susceptibility to glyphosate: 29% are potentially resistant, 7% vary intraspecifically, and 10% are unclassified. Bacteria with sensitive copies of the EPSPS enzyme include *Faecalibacterium, Bifidobacterium,* and *Citrobacter*, whereas *Clostridium, Dorea,* and *Ruminococcus* mostly have resistant sequences. These genera have previously been associated with irritable bowel syndrome [30]. Gastrointestinal issues (such as IBS) and inflammatory conditions have been speculated to arise from gut dysbiosis resulting from glyphosate exposure via the foodstuffs frequently included in a Western diet [31,32].

**Figure 2:**
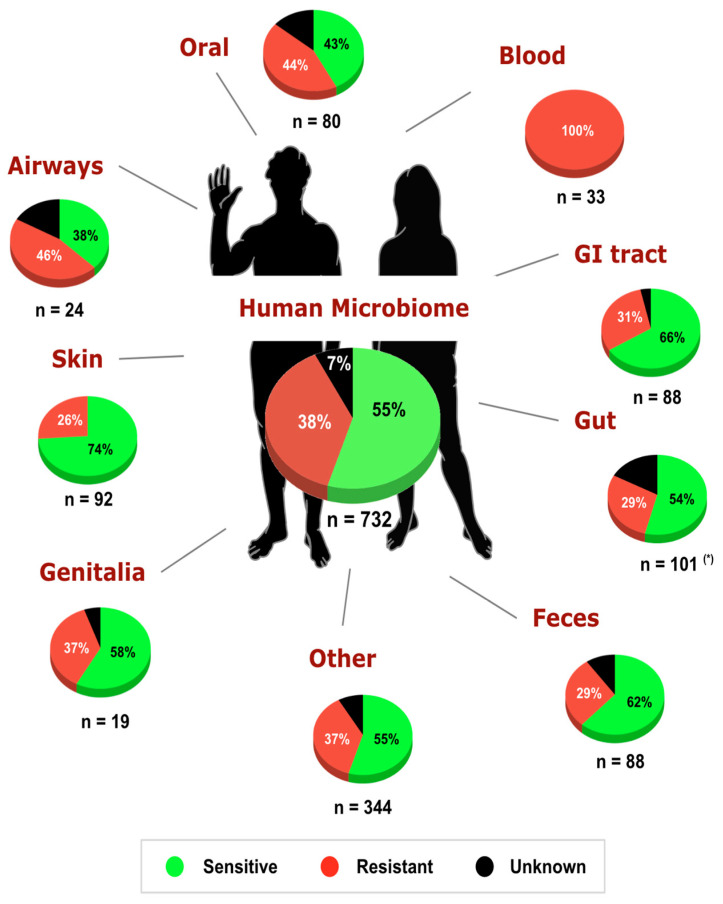
Potential sensitivity to glyphosate in bacteria of the human microbiome project [18]. A total of 732 out of 941 (77.8%) bacterial species from the HMP have at least one copy of the *epsps* gene. Overall, in the human microbiome, the intrinsic sensitivity of bacteria to glyphosate is distributed as 55% sensitive, 38% resistant, and 7% unclassified. (*) Data concerning the sensitivity of gut microbiota were obtained from [6].

Here, with an extended survey of the TSR mechanism for glyphosate in 732 bacteria from the Human Microbiome Project (HMP) [18], we reveal the intrinsic sensitivity of glyphosate in a set of bacterial species of the human microbiome (Figure 2). Analysis of the EPSPS enzyme showed that 55% of bacterial strains isolated from the human body are potentially sensitive to glyphosate, in agreement with our previous study [6], and 77.8% of the strains (732 out of 941) have at least one copy of the *epsps* gene. The breakdown of the dataset by the isolation site of the microbe in the human body revealed differential sensitivity to the herbicide (Supplementary Table S1). A larger proportion of strains inhabiting the oral cavity and airways were intrinsically resistant to glyphosate compared with most other body sites.

The airways are largely dominated by strains from the genera *Neisseria* (including both sensitive and unclassified strains), *Staphylococcus* (all strains are resistant to glyphosate)*,* and *Streptococcus* (all strains are resistant to glyphosate). In the oral cavity, *Streptococcus* strains are dominated by resistant strains, whereas *Prevotella strains* are mostly sensitive to glyphosate. The skin microbiota is dominated by commensal and opportunistically pathogenic species [33] mostly sensitive to the herbicide. For example, the human skin is dominated by *Propionibacterium acnes* (all stains are sensitive to glyphosate) and *Staphylococcus epidermidis* (all strains are resistant to glyphosate). Moreover, 58% of the strains from the urogenital tract and vagina were intrinsically sensitive to the herbicide. Interestingly, all strains isolated from human blood are opportunistic pathogens, in agreement with [34], and intrinsically resistant to glyphosate (*Enterococcus faecalis, Streptococcus sanguinis, Enterococcus faecium*, *Staphylococcus epidermidis, Sporosarcina newyorkensis*, and *Psychrobacter sanguinis*). Associations between microbiome dysbiosis and bloodstream infections (BSIs) have been suggested in immunocompromised [35] and COVID-19 [36] patients. Thus, it is possible that exposure to glyphosate may provide conditions that increase BSI-causing bacteria while decreasing sensitive commensal bacteria.

Recent studies with several animal species suggest that traces of glyphosate in food may lead to alterations of the gut microbiota [6,9,11,26,27,37,38] and changes in the urine metabolome [27,39]. The main elimination route of glyphosate is via urine excretion; thus, glyphosate is frequently found in the urine [40,41]. Glyphosate exposure via nutrition, inhalation, or dermal absorption has been proven by the urinal glyphosate residues detected in several studies [40,42]. Moreover, microbes inhabiting the human oral cavity and airways could be exposed to glyphosate via nutrition and inhalation routes. Dermal exposure, caused mainly by occupational use or the handling of glyphosate [43], may disrupt the skin microbiota.

A healthy human microbiota is defined by its microbial composition, function, dynamics, and ecology [44,45]. Distinctions in glyphosate sensitivity/resistance among bacteria, including the TSR and NTSR mechanisms, may lead to the dysbiosis of normal flora due to differential selection pressure [6,7,27]. The sheer number of intrinsically sensitive bacteria to glyphosate may lead to a potentially emerging disease due to microbial dysbiosis [31]. This includes a possible reduction in bacterial diversity due to a decrease in sensitive bacteria and an increase in resistant and fast-evolving bacteria, which are often pathogenic [46,47,48]. Pathogens tend to have superior stress responses due to their higher adaptiveness under stress conditions; for example, the transition from the environment into their host [46,47]. Thus, glyphosate as a stress factor may reduce bacterial susceptibility, either as a stress response or via mutations and change the bacterial response to antibiotics [12,49,50,51,52,53]. In turn, the heavy use of antibiotics and other chemicals may lead to bacterial co- and cross-resistance to glyphosate and other antimicrobials [8,46,54]. Notably, however, sensitivity towards antimicrobial compounds depends on the given concentration; thus, it is necessary to empirically determine at which level of glyphosate, and GBH, bacteria are resistant or susceptible.

## 4. Conclusions

Hence, does glyphosate affect the human microbiota? Contemporary research points to the herbicide’s potential to disrupt healthy microbiomes, including the human microbiome. Several empirical studies have determined the impact of glyphosate-based products on wild- and host-associated microbiota and called to control the potentially negative consequences on environmental health and sustainability. However, further empirical studies are needed to find a “smoking gun” that determines the effect of glyphosate on the healthy human microbiota. Moreover, additional experimental and epidemiological studies are needed to determine these proposed effects of glyphosate-based products on wild and host-associated microbes to control their potentially negative consequences on human health and ecosystem functions and services.

## Data Availability

EPSPS protein data is available at http://ppuigbo.me/programs/EPSPSClass (accessed on 20 April 2022).

## Supplementary Materials

The following supporting information can be downloaded at: https://www.mdpi.com/article/10.3390/life12050707/s1, Table S1: Potential sensitivity of bacterial species from the human microbiome to glyphosate.
